# Teduglutide treatment during breast feeding in a patient with intestinal failure: a case report

**DOI:** 10.3389/fnut.2025.1567908

**Published:** 2025-07-23

**Authors:** Jan Lillienau, Elin Nilsson, Alexandra Vulcan, Åke Nilsson

**Affiliations:** ^1^Department of Clinical Sciences, Lund University, Lund, Sweden; ^2^Skåne University Hospital, Lund, Sweden

**Keywords:** teduglutide, treatment, breast feeding, wean off, parental nutrition

## Abstract

Successful pregnancy and breast feeding during intravenous treatment for advanced intestinal failure (IF) is possible with careful surveillance and appropriate adjustment of the nutritional regimen. However, experience of GLP-2 agonists in this context is lacking. Here we describe a patient with IF who underwent two successful pregnancies and who used teduglutide during breastfeeding after the second pregnancy, with good effect and without negative side effects.

## Introduction

Pregnancy in patients with advanced intestinal failure (IF) can be managed successfully, with careful surveillance to minimize IF and parenteral nutrition (PN) related complications (1, 2). Pregnancy increases the nutritional demand and enhances hepatic gluconeogenesis, triglyceride production, and VLDL secretion (3). In IF patients interruption of the enterohepatic recirculation increases hepatic bile acid synthesis and triglyceride production, and decreases hepatic fatty acid oxidation, due to the decreased interaction of bile salts with the nuclear farnesoid X receptor (4). The risk for intestinal failure associated liver disease (IFALD) increases with intravenous caloric and lipid load (5). There might thus be an interaction between factors involved in IFALD pathogenesis and factors causing pregnancy related liver disorders as intrahepatic cholestasis of pregnancy, acute fatty liver of pregnancy and HELP syndrome (6). The GLP-2 analog teduglutide reduces the need for intravenous support in IF patients (7, 8), but experience of teduglutide during pregnancy and breast feeding is lacking.

In this report we describe a patient with IF, who underwent two successful pregnancies, and who used teduglutide during breastfeeding of the second child.

## The patient’s intestinal failure

A 30-year-old woman underwent emergency surgery due to small intestinal ischemia caused by strangulation related to intestinal malrotation, during a first-time pregnancy in week 16. The fetus was lost. A high jejunostomy with 35–40 cm of jejunum below ligamentum Treitz remaining was combined with a gastrostomy and an ascendostomy. Soon after surgery the stomal flows were high. Intravenous support amounted initially to 5.5–7 L/d but decreased to about 4 liter/d after intravenous treatment with esomeprazol 40 mg x 2. PN, mostly Smofkabiven plus trace elements and vitamins, was combined with Ringer acetate (RA) and 0.9% NaCl. Strictures in remaining jejunum, which were probably postischemic, set narrow limits for oral nutrition.

Five months after the emergency operation liver tests became abnormal, exhibiting a slowly increasing trend. Values did not improve during a temporary change to fat free PN (Clinimix). The maximum values were for bilirubin 71 umole/L and for ALAT 6.4 ukat/L. Liver biopsy showed no fatty liver, light fibrosis and some cells with balloony degeneration.

10 months after the emergency surgency anastomosis of remaining 35–40 cm jejunum to colon ascendens, stricturoplasty just below ligament of Treitz and close to the jejunal end, and closure of the gastrostomy, were implemented. Soon after this operation the frequency of diarrhea was 10–12 times/day. During the first 6 months after the operation, the diarrhea gradually improved. The need for intravenous support decreased to a stable level of Smofkabiven 986 mL/d plus 0.5 L RA 4–6 days/week. The liver tests rapidly improved and were normal within 3 months.

The patient had one catheter infection soon after the discharge from the hospital after the emergency operation and a second one 4 months later. After the patient had learned to manage her intravenous support autonomously 5 months after the emergency surgery no catheter infection has occurred during the following 9 years. The catheter has, however, been replaced once, after 4 years, due to restricted flow.

## The two pregnancies after the jejuno-colic anastomosis

Details regarding timing, outcome of pregnancies, nutritional support and use of teduglutide are given in Table 1. Half a year after the jejuno-colic anastomosis the patient had stable PN support. Oral food intake was high. Teduglutide treatment was postponed, because pregnancy was planned. The first pregnancy with IF was confirmed 13 months after the reanastomosis surgery, i.e., 23 months after the emergency surgery.

**Table 1:** Course of pregnancies, nutrition support and use of teduglutide.

***Characteristics of the patient’s IF*** During a first pregnancy, in week 16, malrotation related strangulation led to intestinal ischemia requiring extensive resection of small intestine, and to loss of the baby. 10 months later the 35–40 cm long remaining jejunum was anastomosed to colon ascendens, a gastrostomy was closed, and two stricturoplasties on remaining jejunum were carried out. The term “with IF” used below refers to the situation at different time points after this operation.
***First pregnancy with IF*** Confirmed 13 months after the jejuno-colic anastomosis. Normal maternal and fetal development, with Smofkabiven 986 mL/d 6 days a week and in last trimester 1,477 mL four and 986 mL 2 days a week, plus 0–1 L RA/d. Term delivery by acute Cesarian section. Girl. Birth weight 2.9 kg.
***Teduglutide treatment after the first pregnancy with IF*** Treatment (0.05 mg/kg/d) started 1 month after weaning. Rapid improvement of diarrhea. Gradual decrease of PN; 986 mL Smofkabiven plus 0–1 L RA/d from 5 to 2 days a week; with maintained weight stability. Attempt to withdraw all PN led to a gradual decrease in weight from 50–52 kg to 47 kg despite high oral food intake. Intermittent PN was resumed. Abdominal discomfort and pain initial side effect of teduglutide. CT intestine with contrast showed rapid transit to colon and no folds in small intestine.
***Second pregnancy with IF*** After withdrawal of teduglutide due to desire for pregnancy more diarrhea. PN increased to 986 mL Smofkabiven and 0–1 L RA 3–4 times/week. Pregnancy confirmed 56 months after the jejuno-colic anastomosis. 986 mL Smofkabiven/d and 0.5–1 L RA/d 5–7 days a week from ninth gestational week. Normal maternal and fetal development. Delivery via planned Cesarian section. Girl. Birth weight 2.8 kg.
***Teduglutide treatment after the second pregnancy with IF*** Treatment (0.05 mg/kg/d) started 1 month after delivery, given during breastfeeding for 4 months, and then continued after weaning. Decreased frequency of diarrhea. Gradual decrease pf PN from 6 days per week to 3 days per week during breastfeeding. Thereafter low demand for PN, and finally withdrawal of all PN support about 7 years after the jejuno-colic anastomosis, with continued teduglutide treatment. Thus, no PN for the last 2.5 years.

The pregnancy developed normally. Body weight development, obstetric ultrasonography, and laboratory tests were all normal, indicating that the PN adjustment was sufficient to maintain normal fetal growth and maternal weight development. A normal baby was delivered by an acute Cesarian section that was performed because abdominal pain raised suspicion of placental abruption.

Teduglutide treatment was started after weaning. Diarrhea improved, and the PN support could be decreased. After about 3 months of treatment only intermittent intravenous support was necessary. The patient managed without PN for month-long periods. Although long-term weight stability without PN was not achieved the patient thus experienced a good effect of teduglutide.

Planning for another pregnancy teduglutide was withdrawn and PN support was increased. Pregnancy was confirmed 1 year later, 56 months after the jejuno-colic anastomosis. Again, the pregnancy developed normally (Table 1). The child was delivered by planned Cesarian section. The maternal weight increase during both pregnancies was 10 kilos. During the late gestational phases ALAT was moderately raised but normalized soon after delivery.

Teduglutide was reinstated 1 month after the birth of the second child. Breastfeeding continued for another 4 months during teduglutide treatment. As detailed in Table 1 the effect on diarrhea was beneficial and the PN support could be decreased, milk production was sufficient and no side effects of teduglutide were observed. The treatment was continued after weaning. Initially month-long periods without PN support were possible but full weight stability over periods of several months was not achieved without some intermittent PN support. Finally, all PN support could, however, be withdrawn. In May 2025 the patient has not received any PN support for two and a half year, with continued teduglutide treatment (Table 1).

## Discussion

Successful pregnancies are possible in advanced IF (1, 2). Our patient lost her first baby during the emergency, that led to the IF and was early informed that pregnancy would be possible in the new situation, appropriately after the planned jejuno-colic anastomosis.

The risk of complications during pregnancy in IF patients, including complications related to IF such as catheter infections and malnutrition, is increased (1). Teduglutide might decrease this risk, if a larger proportion of the nutrition can be given orally. Experience of teduglutide use during pregnancy is, however lacking, and pregnancy in IF patients can be successfully managed by conventional treatment (1). The outcome for our patient confirms these experiences. The increase in PN during the pregnancy was in the middle of the range reported by Billiauws et al. (1) and the oral food intake was high. As a general feature gastric emptying and intestinal motility are slower during pregnancy, which may explain why our patient experienced improvement of diarrhea during the pregnancies.

After the first child was born and breastfeeding terminated, the patient was treated with teduglutide, with good effect. Although teduglutide was withdrawn before and during the second pregnancy it was reinstated early during breastfeeding. The patient was eager to start again, and we considered it unlikely that teduglutide may be transferred into breast milk or exert harmful effects in the baby or the mother.

Mediation of GLP-2 effects on intestinal blood flow and growth and on gastric emptying are tissue specific. They are mediated via a G-protein coupled receptor that is expressed in enteroendocrine and myofibroblast cells. In these cells GLP-2 increases production of IGF-1, keratinocyte growth factor and epidermal growth factor/Erb ligands (9) which in turn mediate the trophic effects of GLP-2 on the gut mucosa, and other effects on the GI tract. Reported extraintestinal effects of GLP-2 do not include any effect on breast glands (9). In our patient milk production was sufficient. No side effects of teduglutide were observed.

Considering the successful outcome, one may question whether continued use of teduglutide during pregnancy could have offered significant advantages to our patient. As the use of GLP-2 agonists increases, occasional patients may, however, become pregnant during teduglutide treatment or have strong reasons to use it during pregnancy. Gentillini et al. (10) reported successful pregnancy without PN support in an IF patient who had been treated with teduglutide. PN had been withdrawn before the pregnancy. Interestingly, the level of endogenous GLP-2 in this patient was higher than in pregnant women with intact intestine.

Animal experimental studies of teduglutide effects during pregnancy are lacking. A highly relevant background observation is, however, that in pig fetuses GLP-2 was undetectable in plasma as late as day 98 but was present in high concentration at full term day 115, and exogenous GLP-2 did not affect fetal intestinal growth (11). Natural maternal GLP-2 is thus not transferred to fetal blood and does not exert effects in the fetus. If teduglutide acts like natural GLP-2 in this respect, one may expect that teduglutide administration during pregnancy will not affect the fetus.

Severe liver reactions were not observed in the 15 patients studied by Billiauws et al. (1). Our patient only exhibited a modest reversible increase in liver tests late during pregnancy. Findings thus do not indicate that IF and pregnancy related factors exhibit harmful interactions in development of liver pathology.

In summary teduglutide may help mothers with IF even during the breastfeeding period when nutritional demands are increased.

## Data Availability

The original contributions presented in the study are included in the article/supplementary material, further inquiries can be directed to the corresponding author.
